# Historical Nonsuicidal Self‐Injury and Current Coping Strategies: The Role of Emotion Regulation

**DOI:** 10.1002/pmh.70035

**Published:** 2025-08-20

**Authors:** Hannah Bradley, Faith Orchard, Faith Matcham

**Affiliations:** ^1^ School of Psychology University of Sussex Brighton UK; ^2^ University of East Anglia Norwich UK

**Keywords:** adaptive coping, emotion regulation, maladaptive coping, nonsuicidal self‐injury

## Abstract

Supporting individuals to develop adaptive coping strategies is critical to improve health and wellbeing. A history of nonsuicidal self‐harm (NSSI) is linked with increased risk of maladaptive coping strategies. We aimed to identify whether emotion regulation (ER) mediated the relationship between historical NSSI behaviours and coping strategy use in adults from the general population. Participants reported their lifetime frequency of NSSI; then they completed self‐report measures of emotion regulation, maladaptive coping strategy use (behavioural disengagement, self‐blame and substance use) and adaptive coping strategy use (active coping, instrumental support use and emotional support use). Regression analyses investigated relationships between historical NSSI and coping strategies, with mediation analysis testing for the mediating role of ER. A total of 303 individuals participated: 180 with a history of NSSI and 123 without. Results indicated that poor emotion regulation mediated the relationship between having a history of NSSI and the use of maladaptive coping strategies; however, there was no notable association between adaptive coping strategies and historical NSSI. These findings highlight the necessity for ER development in people with a history of NSSI and suggest important avenues for future research, including exploring the role of ER to support NSSI cessation.

## 1 Introduction

Mental health problems cost the United Kingdom £117.9 billion in 2019, largely due to loss of productivity in employment and informal care costs (McDaid and Park 2022). There is a well‐established connection between experiencing high levels of stress and increased risk of poor mental health. Understanding and improving stress coping mechanisms may have important implications for improving mental health outcomes (Thoits 2010). Richard Lazarus (1966) defined coping as a way of cognitively or behaviourally responding to demands that are viewed as taxing for the individual. Our appraisal of these demands can be influenced by the effectiveness of our coping response (Lazarus and Folkman 1984). Coping strategies are often divided into adaptive (improving wellbeing and resilience through healthy behaviours) or maladaptive (hindering wellbeing and resilience through unhealthy behaviours) (Dijkstra and Homan 2016). Maladaptive coping strategies have been associated with lower psychological wellbeing (Lehavot 2012). Whereas adaptive coping strategies may build resilience to stress (Earnshaw et al. 2015). Understanding the contributing and consequential factors for the use of certain coping strategies can inform future research on improving mental health‐related outcomes and reduce its economic impact.

Although there are over 400 identified subtypes of both adaptive and maladaptive coping responses (Skinner et al. 2003), there is growing evidence highlighting the importance of nonsuicidal self‐injury (NSSI; Nock 2009), behavioural disengagement (Carver et al. 1989), self‐blame (Ullman et al. 2014), substance misuse (Carver et al. 1989), active coping (Flouri and Mavroveli 2013), emotional support seeking (Döveling 2015) and instrumental support seeking (Lobel et al. 2014) as particularly relevant for understanding mental health problems (Budimir 2021; Gurvich et al. 2021). NSSI is defined as the direct and deliberate harm caused to one's own body without suicidal intent (Nock 2009) and has been found to be strongly associated with other maladaptive coping strategies (Sorgi et al. 2021), such as behavioural disengagement (Nock and Mendes 2008) and self‐blame (Kelada et al. 2018). Both cannabis and alcohol use are reported to have positive relationships with NSSI (Bresin and Mekawi 2022; Escelsior et al. 2021; Turner et al. 2022), with self‐harm during adolescence being a predictor for future substance use in young adulthood (Moran et al. 2015).

Active coping occurs when individuals make direct and effortful attempts to remove or respond to the stressor (Carver et al. 1989). Emotional support seeking involves individuals seeking emotional support and understanding from others (Carver et al. 1989). Instrumental support seeking involves individuals seeking advice or assistance regarding the stressor (Carver et al. 1989). Active coping is reported to have a negative relationship with NSSI, as well as with NSSI frequency (Chapman et al. 2014). Reports in older adults show that their perceptions of never receiving emotional support are positively related to hospitalizations for self‐harm, and that seeking support predicts less self‐harm in adolescent samples, although this was specific to social support rather than emotional (Shaw et al. 2021). Similarly, in adolescent samples, instrumental help‐seeking for self‐harm is uncommon, even though help is likely wanted (Doyle et al. 2015; Michelmore and Hindley 2012). Relationships between NSSI and adaptive coping strategies have received considerably less attention despite their possible effects on resilience (Earnshaw et al. 2015).

Emotion regulation (ER) may be a crucial factor in explaining the link between NSSI and coping. ER is the ability to respond healthily to emotions to control their duration, intensity and expression (Gratz and Roemer 2004; Rolston and Lloyd‐Richardson 2016). The ability‐based model of ER proposes that there are abilities that help facilitate and create the potential for ER (Gratz and Roemer 2004; Naragon‐Gainey et al. 2017). Maladaptive coping strategies may only temporarily regulate emotions, doing so superficially and without full resolution of the emotion, often impacting negatively on life experiences (Midkiff et al. 2018). Research has found that ER is associated with active coping (Flouri and Mavroveli 2013), emotional support seeking (Döveling 2015), and instrumental support seeking (Flouri and Mavroveli 2013; Lobel et al. 2014), and difficulties in emotion regulation (DER) are associated with behavioural disengagement (Kahn et al. 2022), self‐blame (Ullman et al. 2014), and substance use (Garke et al. 2021; Weiss et al. 2022). However, child and adolescent samples have dominated much of the coping literature (e.g., Doyle et al. 2015; Flouri and Mavroveli 2013; Garke et al. 2021; Michelmore and Hindley 2012; Nock and Mendes 2008).

Reasons behind engaging in NSSI can vary from individual to individual (Edmondson et al. 2016), but most often its function serves to reduce negative affect through ER (Brereton and McGlinchey 2020; Hasking et al. 2017). The notion that ER is the primary driving force of NSSI has been suggested and included in models, such as the four‐factor model of NSSI by Nock and Prinstein (2004). Davis et al. (2014) asked adult participants with and without a history of deliberate self‐harm (DSH) to watch a sad film clip and self‐report negative emotions with and without implementing reappraisal as an emotion regulation ability. Their results demonstrated DER is correlated with having a history of DSH; however, there was no distinction between those who had recently engaged in DSH and those who no longer engage in DSH. Evidence has suggested these two groups have significant differences in their ER. For example, Zielinski et al. (2018) collected self‐report measures of NSSI recency and DER and found that those who are currently self‐injuring display greater DER than those who have engaged in NSSI previously. However, again, they miss important comparisons with individuals who have no history of NSSI. Such research reports individuals with current or past NSSI engagement experience more DER than those without a history (Anderson and Crowther 2012), and that these individuals without a history of NSSI have greater ER instead (Nielsen et al. 2017). There have also been further distinctions within NSSI involving frequency. Researchers have explored this relationship and share similar findings showing greater DER is associated with increased NSSI/self‐harm frequency (Davis et al. 2014; Zielinski et al. 2018).

The current study addresses the complex interplay between NSSI, ER and coping (Clapham and Brausch 2022), offering an original contribution to the literature by exploring the mediating role of ER between NSSI and both maladaptive and adaptive coping strategies (Clapham and Brausch 2022). It is beneficial to explore potential mediational effects ER may have in the relationship between historical NSSI behaviours and current maladaptive and adaptive coping strategies to inform future research and mental health support. While prior work has often focused on clinical or adolescent samples, this study extends the literature by assessing whether these relationships persist in a community‐based context. Results may differ from those in clinical samples due to underlying psychiatric morbidity and high severity symptom experiences in clinical populations. Some studies have included control groups in clinical samples for NSSI, but often these have low sample sizes (Tschan et al. 2015). Therefore, increased information about NSSI in the general population is needed. Moreover, the proposed mediation model in the current paper adds mechanistic insight into how emotional functioning might sustain maladaptive coping, even after NSSI cessation. These distinctions enhance the applicability of findings to early intervention and preventative care settings.

It is hypothesized that:

1. The presence of historical NSSI and higher frequency of NSSI will predict increased use of the maladaptive coping strategies: behavioural disengagement, self‐blame and substance use.
2. The absence of historical NSSI will predict increased use of the adaptive coping strategies: active coping, emotional support seeking and instrumental support seeking.
3. The presence of historical NSSI and higher frequency of NSSI will be associated with poorer ER.
4. ER will mediate the relationship between historical NSSI behaviours and both maladaptive and adaptive coping strategies.

## 2 Methods

### 2.1 Sample

In this cross‐sectional study, data were collected via Qualtrics surveys through opportunity sampling from social networking sites, University of Sussex staff and student circulars, and leading mental health organizations. Recruitment lasted from April to December 2022. This study was ethically approved by the Sciences & Technology Cross‐Schools Research Ethics Committee at the University of Sussex (ER/FM409/2).

Individuals needed to meet the following criteria to participate: reside in the United Kingdom, be at least 18 years old, have not engaged in self‐injury within the past 12 months, and not be distressed by topics of NSSI or substance use. It was important for participants to have not self‐injured within the past 12 months as people's ER has been found to significantly differ depending on the recency of their last self‐injury (Zielinski et al. 2018). Power calculation was based on multiple linear regression conducted to detect an association between the presence of historical NSSI and the use of adaptive or maladaptive coping strategies. Assuming an alpha of 0.05, power at 80%, and adjustment for two covariates, a sample size of at least 80 people per NSSI group (with/without a history of NSSI) would be required (Faul et al. 2007).

### 2.2 Measures

#### 2.2.1 Demographics

Participants reported on their age and gender as categorical variables. They selected their age from the following categories: 18–29 years old, 30–39 years old, 40–49 years old, 50–59 years old, and 60 years or older. Participants indicated their gender by choosing from the following categories: Female, Male, Nonbinary, Other and Prefer not to say.

#### 2.2.2 Nonsuicidal Self‐Injury

To assess NSSI, the Deliberate Self‐Harm Inventory (DSHI; Gratz 2001) was used. The DSHI is a 17‐item questionnaire involving an index question and five follow‐up questions for each item. Items in the questionnaire are methods of DSH that are implemented into the index question. As methods of NSSI were not being explored, all items were combined to just one (‘hurt yourself?’). Responses were either ‘Yes’ or ‘No’ A response of ‘Yes’ resulted in further follow‐up questions.

Firstly, addressing the recency of participants' last NSSI episode, options for this question were ‘Within the past 12 months’ and ‘Over 12 months ago’. Participants indicating self‐injury within the past 12 months were excluded from the analysis. The second follow‐up question asked about frequency of NSSI. Options for this were limited to fixed choice ranges (‘1–5 times’, ‘6–10 times’, ‘11+ times’) to avoid extremely high self‐reported frequencies (Kapatais et al. 2022). This was implemented as those who have self‐injured many times are more likely to be inaccurate in their estimation of total episodes compared with those who have self‐injured fewer times (Nielsen et al. 2016). The DSHI has been found to have adequate test re‐test reliability over a mean period of 3.3 weeks (φ = 0.68, *p* < 0.001; Gratz 2001), and adequate construct, convergent, and discriminant validity (Gratz 2001). In this study, it had good internal consistency (*α* = 0.87).

#### 2.2.3 Emotion Regulation

Emotion regulation was measured using the Difficulties in Emotion Regulation Scale Short Form (DERS‐SF; Kaufman et al. 2016). The DERS‐SF comprises of 18 items loaded onto six subscales. Each item is scored from 1 (*Almost never (0%–10%)*) to 5 (*Almost always (91%–100%)*), with exception for the reverse‐coded items. Items were summed to create a total score (ranging from 18 to 90) whereby a higher score represented poorer emotion regulation. The DERS‐SF has comparable concurrent validity to its original DER scale which demonstrated good test–retest reliability and adequate construct and predictive validity (Gratz and Roemer 2004; Kaufman et al. 2016). In this study, it had good internal consistency (*α* = 0.84).

#### 2.2.4 Maladaptive and Adaptive Coping Strategies

To measure both maladaptive and adaptive coping strategies, the Brief Coping with Problems Experienced inventory (COPE; Carver 1997) was used. The Brief COPE inventory includes 14 scales with two items in each scale. Three scales were chosen for maladaptive coping strategies: behavioural disengagement, self‐blame and substance use. Another three scales were chosen for adaptive coping strategies: active coping, using emotional support and using instrumental support. Each item is scored from 1 (*I haven't been doing this at all*) to 4 (*I've been doing this a lot*). Items were summed to create a total score for each scale (ranging from 2 to 8) whereby a higher score represented more use of that coping strategy. Each scale had good internal consistency: behavioural disengagement (*α* = 0.76), self‐blame (*α* = 0.80), substance use (*α* = 0.96), active coping (*α* = 0.78), using emotional support (*α* = 0.86), and using instrumental support (*α* = 0.90). The Brief COPE inventory had comparable internal reliability to its original COPE inventory which demonstrated adequate convergent and discriminant validity (Carver et al. 1989; Carver 1997).

### 2.3 Data Analysis

All statistical analyses were conducted using IBM SPSS Statistics Version 28, with a significance threshold set at *p* < 0.05. Preliminary checks, alongside reference to the central limit theorem (Field 2018), confirmed that the assumptions of the linear model were satisfied, allowing analyses to proceed as planned.

To address Hypothesis 1, linear regression analyses were performed. Separate models were computed with NSSI history and NSSI frequency as predictor variables, and behavioural disengagement, self‐blame and substance use as outcome variables. All models were adjusted for age and gender.

For Hypothesis 2, linear regression analyses were similarly conducted, with NSSI history entered as the predictor variable and active coping, instrumental support and emotional support use as outcome variables. These models were also adjusted for age and gender.

Hypothesis 3 was tested using independent samples *t*‐tests, with NSSI history and NSSI frequency as the predictor variables and difficulties in emotion regulation (DER) as the outcome variable.

Finally, Hypothesis 4 was examined in two steps. First, Pearson's correlations with confidence intervals were calculated to assess the relationships between DER and all coping strategy variables. Subsequently, mediation analyses were conducted using the PROCESS macro (Hayes 2017), with NSSI history as the predictor, maladaptive coping strategies as the outcome variables, and emotion regulation (ER) as the mediator. Mediation analyses followed the criteria outlined by Baron and Kenny (1986), requiring significant associations between the predictor, mediator and outcome variables. The significance of mediation effects was determined using bootstrapped confidence intervals, with significance indicated by intervals that did not contain zero (Field 2018).

## 3 Results

### 3.1 Participant Characteristics

Recruitment resulted in 407 potential participants. Fifteen (3.69%) were excluded due to incomplete data, and 33 (8.11%) due to having reported NSSI within the past 12 months (making them ineligible to participate), leaving a final sample of 303 (74.45%) participants; 180 with a history of NSSI and 123 without a history of NSSI. Only 24 respondents reported having engaged in NSSI six‐to‐ten times. These responses were combined with the responses from those reporting NSSI one‐to‐five times to create a new group consisting of those having self‐injured one‐to‐ten times.

Table 1 presents the descriptive statistics for all study variables. In comparison with those with no history of NSSI, those with a history of NSSI were significantly more likely to be in the youngest age group (18–29 years), had higher DER, increased behavioural disengagement, self‐blame and substance use.

**TABLE 1:** Descriptive statistics with comparisons between those with no history of NSSI and those with a history of NSSI.

		Total sample	No history of NSSI	History of NSSI
Total, *N* (%)		303 (100)	123 (40.60)	180 (59.40)
Age, *N* (%)	18–29 years	276 (91.10)	109 (88.62)**	167 (92.78)**
	30–39 years	11 (3.60)	4 (3.25)	7 (3.89)
	50–59 years	13 (4.30)	8 (6.50)	5 (2.78)
	60+ years	3 (1.00)	2 (1.62)	1 (0.56)
Gender, *N* (%)	Female	237 (78.20)	101 (82.11)	136 (75.56)
	Male	44 (14.50)	22 (17.89)	22 (12.22)
	Nonbinary	17 (5.60)	0 (0.00)	17 (9.44)
	Other	2 (0.70)	0 (0.00)	2 (1.11)
	Prefer not to say	3 (1.00)	0 (0.00)	3 (1.67)
NSSI frequency, *N* (%)	1–10 times	89 (29.40)	—	89 (49.44)
	11+ times	91 (30.00)	—	91 (50.56)
DER, *M* (SD)		52.91 (10.80)	49.16 (9.07)***	55.48 (11.16)***
Behavioural Disengagement, *M* (SD)		3.62 (1.52)	3.37 (1.48)*	3.78 (1.53)*
Self‐blame, *M* (SD)		5.66 (1.81)	5.24 (1.79)***	5.94 (1.77)***
Substance use, *M* (SD)		3.45 (1.90)	2.96 (1.60)***	3.78 (2.02)***
Active coping, *M* (SD)		5.55 (1.55)	5.52 (1.44)	5.57 (1.62)
Using instrumental support, *M* (SD)		5.03 (1.94)	5.15 (1.92)	4.95 (1.96)
Using emotional support, *M* (SD)		5.27 (1.84)	5.41 (1.86)	5.18 (1.83)

*p < 0.05, **p < 0.01, ***p < 0.001, two‐tailed significance tests.

Abbreviations: DER = difficulties in emotion regulation, tests of significant difference between no history/history of NSSI; NSSI = nonsuicidal self‐injury.

### 3.2 Hypothesis Testing

Hypothesis 1 The presence of historical NSSI, and higher frequency of NSSI, will predict increased use of maladaptive coping strategies.

Results of the linear regressions examining the relationships between a history of NSSI, frequency of NSSI, and the maladaptive coping strategies are reported in Table 2. There were significant relationships between NSSI history and all three maladaptive coping strategies when both unadjusted and adjusted for age and gender from the *p*‐values and 95% CIs. The *b*‐values for the relationships between NSSI history and the maladaptive coping strategies show that in comparison with those with no history of NSSI, a history of NSSI increases these maladaptive coping strategies by less than one point on its 4‐point Likert scale. There were no significant relationships between NSSI frequency and the maladaptive coping strategies in either adjusted or unadjusted models. The *b*‐values for the relationships between NSSI frequency the maladaptive coping strategies show that compared with those with a smaller lifetime frequency of NSSI (one‐to‐ten times), those with a higher lifetime frequency of NSSI (11+ times) show increased use of the maladaptive coping strategies by less than one point on its 4‐point Likert scale. The results from these regression analyses show partial support for Hypothesis 1.

Hypothesis 2 The absence of historical NSSI will predict increased use of adaptive coping strategies.

**TABLE 2:** Linear regression results for NSSI behaviours predicting use of maladaptive coping strategies.

Predictors		Behavioural disengagement					Self‐blame					Substance use				
		*b* (95% CI)	*SE B*	*β*	*p*	*r* ^2^	*b* (95% CI)	*SE B*	*β*	*p*	*r* ^2^	*b* (95% CI)	*SE B*	*β*	*p*	*r* ^2^
*Unadjusted*																
NSSI History	No (ref)	—	—	—	—	—	—	—	—	—	—	—	—	—	—	—
	Yes	0.41 (0.06, 0.76)	0.18	0.13	0.021	0.02	0.70 (0.29, 1.10)	0.21	0.19	< 0.001	0.04	0.82 (0.39, 1.25)	0.22	0.21	< 0.001	0.05
NSSI Frequency	1–10 times (ref)	—	—	—	—	—	—	—	—	—	—	—	—	—	—	—
	11+ times	0.34 (−0.13, 0.80)	0.24	0.11	0.157	0.01	0.07 (−0.47, 0.62)	0.28	0.02	0.791	0.00	0.16 (−0.46, 0.78)	0.32	0.04	0.617	0.00
*Adjusted* ^a^																
NSSI History	No (ref)	—	—	—	—	—	—	—	—	—	—	—	—	—	—	—
	Yes	0.38 (0.03, 0.73)	0.18	0.12	0.036	0.02	0.64 (0.23, 1.05)	0.21	0.17	0.003	0.05	0.87 (0.43, 1.30)	0.22	0.22	< 0.001	0.05
NSSI Frequency	1–10 times (ref)	—	—	—	—	—	—	—	—	—	—	—	—	—	—	—
	11+ times	0.34 (−0.13, 0.81)	0.24	0.11	0.153	0.02	0.08 (−0.46, 0.62)	0.27	0.02	0.778	0.02	0.15 (−0.46, 0.77)	0.31	0.04	0.627	0.01

Abbreviation: NSSI = nonsuicidal self‐injury.

^a^ Adjusted for age and gender.

Results of the linear regressions examining the relationships between NSSI history and the adaptive coping strategies are reported in Table 3. No significant relationships between a history of NSSI and the adaptive coping strategies were found. The *b*‐values for the relationships between NSSI history and instrumental support use and emotional support use show that in comparison with those with no history of NSSI, a history of NSSI decreases the use of these adaptive coping strategies by less than one point on its 4‐point Likert scale. The *b*‐values for the relationship between NSSI history and active coping show opposite results in that a history of NSSI, compared with no history, increases the use of this strategy by less than one point on its 4‐point Likert scale. These are unsubstantial changes and overall show no support for Hypothesis 2.

**TABLE 3:** Linear regression results for NSSI history predicting use of adaptive coping strategies.

Predictors		Active coping					Instrumental support					Emotional support				
		*b* (95% CI)	*SE B*	*β*	*p*	*r* ^2^	*b* (95% CI)	*SE B*	*β*	*p*	*r* ^2^	*b* (95% CI)	*SE B*	*β*	*p*	*r* ^2^
*Unadjusted*																
NSSI history	No (ref)	—	—	—	—	—	—	—	—	—	—	—	—	—	—	—
	Yes	0.05 (−0.31, 0.41)	0.18	0.02	0.775	0.00	−0.20 (−0.65, 0.24)	0.23	−0.05	0.370	0.00	−0.23 (−0.65, 0.20)	0.22	−0.06	0.289	0.00
*Adjusted* ^a^																
NSSI history	No (ref)	—	—	—	—	—	—	—	—	—	—	—	—	—	—	—
	Yes	0.06 (−0.31, 0.42)	0.19	0.02	0.761	0.00	−0.13 (−0.59, 0.32)	0.23	−0.03	0.569	0.02	−0.16 (−0.59, 0.27)	0.22	−0.04	0.470	0.02

Abbreviation: NSSI = nonsuicidal self‐injury.

^a^ Adjusted for age and gender.

Hypothesis 3 The Presence of Historical NSSI, and Higher Frequency of NSSI, Will be Associated With Poorer ER.

The participants who have a history of NSSI compared with the participants who do not demonstrate significantly poorer ER, *t*(301) = −5.21, *p* < 0.001, with a represented medium effect of *d* = 0.61. Furthermore, participants who have a higher frequency of NSSI compared with participants who have a lower frequency also demonstrate significantly poorer ER, *t*(178) = −2.79, *p* = 0.006, with a represented smaller effect of *d* = 0.43. These results suggest that having a history of NSSI and increased frequency of NSSI is related to poorer ER, providing support for Hypothesis 3.

Hypothesis 4 ER Will Mediate the Relationship Between Historical NSSI Behaviours and Coping Strategies.

Results of the correlations examining the relationships between DER and the coping strategies are reported in Table 4. It was found that DER has a significant, positive relationship with behavioural disengagement and substance use with medium effect sizes. There was also a significant, positive relationship between DER and self‐blame which represents a large effect. DER had a close to zero relationship with both instrumental support use and emotional support use, showing no significant relationship. However, DER had a small, positive and significant relationship with active coping. These correlations provide partial support for Hypothesis 4.

**TABLE 4:** Pearson's correlations between the study variables.

Variable	DER
Behavioural disengagement	0.38** [0.30, 1.00]
Self‐blame	0.54** [0.47, 1.00]
Substance use	0.36** [0.27, 1.00]
Active coping	−0.15* [−1.00, −0.06]
Instrumental support	0.01 [−0.08, 1.00]
Emotional support	−0.06 [−1.00, 0.03]

*Note:* 95% CIs reported in brackets. *p < 0.01, **p < 0.001, one‐tailed significance tests.

Abbreviation: DER = difficulties in emotion regulation.

**FIGURE 1:**
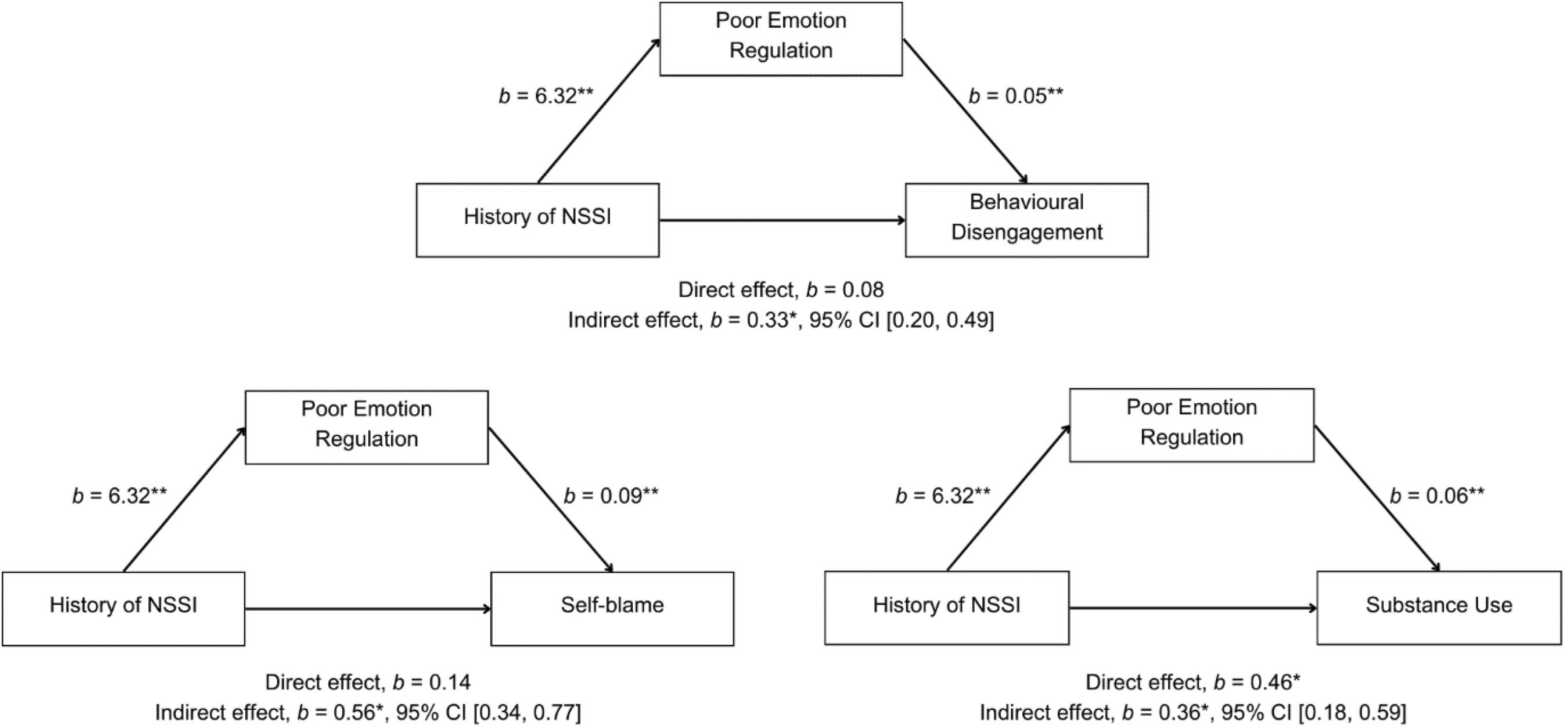
Model illustrating the relationship between NSSI history and maladaptive coping strategies, mediated by poor emotion regulation (ER). The confidence interval for the indirect effect is a bias‐corrected and accelerated bootstrapped CI based on 5000 samples; *p < 0.05, **p < 0.001.

Figure 1 reports results from mediation analyses where NSSI history predicts the maladaptive coping strategies, mediated by poor ER. NSSI history significantly predicted poorer ER, and poorer ER significantly predicted behavioural disengagement, self‐blame and substance use whilst controlling for NSSI history. There was a bigger variance of the model explained by poorer ER on behavioural disengagement (14.70%), self‐blame (28.95%) and substance use (14.10%) than there was for NSSI history on behavioural disengagement (1.76%), self‐blame (3.58%) and substance use (4.48%). The direct effect of NSSI history on substance use whilst controlling for poor ER remained significant whereas its effect on behavioural disengagement and self‐blame resulted in no significant results. The mediation analyses produced significant indirect effects of NSSI history on behavioural disengagement, self‐blame and substance use through poor ER. These results provide partial support for Hypothesis 4.

## 4 Discussion

This study expanded previous research to examine the mediational effects of ER between NSSI and coping strategies and include more insight into these topics within the adult general population. We identified significant relationships between having a history of NSSI and the maladaptive coping strategies. Having a history of NSSI, but not increased frequency of NSSI, increased the likelihood of using behavioural disengagement, self‐blame and substances to cope. These results support previous literature finding NSSI history to relate to these maladaptive coping strategies (Bresin and Mekawi 2022; Escelsior et al. 2021; Kelada et al. 2018; Moran et al. 2015; Nock and Mendes 2008; Turner et al. 2022). Understanding why increased NSSI frequency does not increase the use of these maladaptive coping strategies may be explained by how this study categorized NSSI frequency. The Diagnostic and Statistical Manual of Mental Disorders (DSM‐5; American Psychiatric Association 2022) included nonsuicidal self‐injury disorder (NSSID) as a criterion set requiring further research. Their proposed frequency to meet a NSSID diagnosis is the engagement in NSSI over five or more days. Although a higher threshold may be warranted (Andover 2014; Washburn et al. 2015), it suggests that the NSSI frequency categories within this study were not able to differentiate between historically clinical and subclinical groups of people who engaged in NSSI due to overlap, particularly within the group that selected their engagement as ‘1–10 times’ (Muehlenkamp et al. 2017). Future research investigating NSSI frequency should consider this in their design.

We did not find evidence of an association between having a history of NSSI and using adaptive coping strategies. These results contradict our prediction and previous findings suggesting that NSSI is related to less use of these adaptive coping strategies (Chapman et al. 2014; Doyle et al. 2015; Michelmore and Hindley 2012; Shaw et al. 2021). There are noteworthy parallels and differences between our findings and those of Chapman et al. (2014), who studied incarcerated women. In their work, active coping was inversely associated with NSSI history. While our study found no significant associations between NSSI history and adaptive coping strategies, the contrast may be due to contextual differences: the stressors, environmental constraints and other available coping resources in prison settings may alter the functionality and accessibility of coping mechanisms (Luke et al. 2021). Moreover, Chapman et al. (2014) did not assess ER directly, which limits the mechanistic interpretation. Our mediation model provides additional insight into the processes underpinning coping behaviours in those with a history of NSSI.

Moving from adolescence to young adulthood comes with maturation of neurological systems associated with improved judgement and management of behaviours, as well as increases in social and emotional stability (Kim and Hur 2023; Siegler et al. 2020; Whitlock et al. 2015). This current study's results demonstrate why it is important not to assume generalizability from other similar studies' significant findings when sampling from, or applying results to, different populations. However, the lack of demographic diversity within this current study, being largely female and young adults, will need to be addressed in future research.

We found that having a history of NSSI, as well as a lifetime frequency of 11 times or more for NSSI, was related to poorer ER, supporting Hypothesis 3. These findings support previous evidence in the field (Adrian et al. 2019; Anderson and Crowther 2012; Clapham and Brausch 2022; Davis et al. 2014; Kapatais et al. 2022; Nielsen et al. 2017; Zielinski et al. 2018). It is thought that the potential for ER has underlying dispositional abilities which could explain why DER persists beyond NSSI cessation and is occurring within this sample (Gratz and Roemer 2004; Wolff et al. 2019). However, it also appears poorer ER presents more in those with a higher lifetime frequency of NSSI. Alongside its potential dispositional nature, Daukantaité et al. (2021) reported in a longitudinal study that repetitive NSSI during adolescence predicted DER ten years later. Furthermore, there may also be differences between these groups as to their perceptions of their own ER abilities. Mettler et al. (2021) found those with a history of NSSI to have reported greater DER than those without a history of NSSI. However, interestingly, both groups experienced similar negative affect elicitation in response to an in vivo mood induction task, perhaps suggesting their self‐reported differences could be due to subjective interpretations of their emotional experiences and their perceived sensitivity to intense feelings rather than a reflection of their actual coping abilities (Mettler et al. 2021). Although this current study did not examine the DERS‐SF subscales, there are links between historical NSSI and the intensity of emotional experiences and limited access to ER strategies (Anderson and Crowther 2012). Further research is necessary to explore whether differences in ER can be attributed to perceptions of one's own ER abilities between these groups and to understand the reasons behind it.

It is also important to consider the potential developmental trajectory of emotional regulation. The finding that younger participants were more likely to report ER difficulties may reflect normative age‐related changes, as previous research shows ER abilities improve with maturity (Compas et al. 2017). However, our models statistically adjusted for age, suggesting that the observed associations with NSSI history reflect more than developmental stage. Nonetheless, longitudinal research is warranted to determine whether these ER difficulties persist or diminish over time.

The main findings of this study from Hypothesis 4 highlighted that poor ER mediates the relationship between historical NSSI and maladaptive coping. This suggests that having a history of NSSI is associated with poor ER, which in turn predicts the use of behavioural disengagement, self‐blame and substances as ways of coping. This supports existing literature with similar findings (Flouri and Mavroveli 2013; Garke et al. 2021; Ullman et al. 2014; Weiss et al. 2022). However, it also suggests DER is not associated with using instrumental support nor emotional support. These findings were not expected, considering previous research to the contrary (Bailey et al. 2016; Criss et al. 2016; Döveling 2015; Flouri and Mavroveli 2013; Lobel et al. 2014; Tambling et al. 2023). Poorer ER is associated with greater mental health difficulties, and healthy ER is associated with less (Aldao et al. 2010; Barlow et al. 2017; Ludwig et al. 2019). The relevance of poor ER lies within its contribution to the development, maintenance and treatment of mental health difficulties, with these relationships potentially varying by type of psychopathology (Aldao et al. 2010; Berking and Wupperman 2012). Others have also argued this relationship is bidirectional, suggesting DER can be both symptomatic and predictive of mental health difficulties (Dawel et al. 2021). Mental health also has strong links to coping. The presence of psychopathology has consistent associations with increased use of maladaptive coping strategies compared with adaptive coping strategies and may also occur because of maladaptive coping (Cohen et al. 2020; Compas et al. 2017; Richardson et al. 2021). The role of psychopathology shows likely confounding effects between ER and coping and may explain the findings reported in the present study. Further research should take care to consider including potentially confounding variables such as this into their study to ensure true effects of ER on coping are being observed.

### 4.1 Strengths, Limitations and Future Directions

This study represents a unique examination of the interplay between NSSI, ER and coping in an adult population. Other research has often related each of these variables together but has rarely tried to combine them all into one model. It furthermore expanded this research to adults from the general population, who are typically overlooked compared to paediatric and clinical samples. We used well‐validated measures of psychological constructs of coping, ER and NSSI. Finally, recruitment via social media provided the potential for a wide variety of demographic characteristics in participants, and its use helped utilize limited recruitment time efficiently (Allsworth 2015; Bartell 2014).

Despite the strengths of our approach, the cross‐sectional design and the correlational analyses of this study limit causal and longitudinal inferences from the results (Setia 2016). It cannot be determined whether poorer ER associated with those with a history of NSSI indicates: (a) any kind of increase or decrease in their ability for ER from when they were currently engaging in NSSI (residual symptom), or (b) a long‐term pattern of poor ER which reflects subgroups of those who stop self‐injuring over time (premorbid trait; Brown et al. 2007). Future research is needed to investigate the path of ER during current NSSI and after its cessation through longitudinal designs to better understand this relationship and examine potential causality. It is important to highlight that individuals who had engaged in self‐injury within the past 12 months were excluded from this study. While this criterion was implemented to minimize participant distress and ensure ethical compliance, it may have resulted in a sample that differs in clinically meaningful ways from currently self‐harming individuals. Recent evidence (Zielinski et al. 2018) indicates that ER difficulties can differ significantly between current and former NSSI users. Therefore, the exclusion of people with recent self‐harm likely constrained variability in ER and coping resources, potentially attenuating some associations. Future research, including both current and former NSSI participants, is needed to understand temporal changes in ER and coping strategies.

Furthermore, the present study is limited in generalizing its findings to the investigation of NSSI, ER and coping in young, female adults. Gender imbalance in NSSI research is an unfortunately common limitation which brings concern, as there are reported gender differences in NSSI behaviours, as well as in ER and coping (Bresin and Schoenleber 2015; Cipriano et al. 2017; Moritz et al. 2016; Zimmermann and Iwanski 2014). Future research is needed to ensure a balance of genders within these groups and a sample representing the ages of the general population. In addition to the previously discussed future directions, it would benefit the literature to continue exploring NSSI as a predictor variable. There is further scope to understand NSSI from its onset, duration, last episode, methods, severity of injuries and cessation, and its implications for not only ER and current coping but also other related outcomes. Finally, this study investigated only a few coping strategies, and so there is room to continue exploring NSSI and ER with other strategies.

### 4.2 Clinical Implications

The results from this study add preliminary support for employing Dialectical Behaviour Therapy skills training (DBT‐ST) for individuals whose behaviours cause mental distress and physical harm, as people with a history of NSSI are more likely to have poorer ER, which results in coping maladaptively than those without a history of NSSI. DBT‐ST aims to replace maladaptive behaviours with goal‐oriented behaviours through four primary skills, including ER skills (Cavicchioli et al. 2021; Eist 2015). In a recent review by Warner and Murphy (2022), DBT‐ST was generally found to reduce substance use and increase ER for people with substance use disorder. Although their review did not provide firm evidence for the acceptability and feasibility of DBT‐ST, research such as this present study continues to demonstrate the need for ER enhancement in treatment options, especially for those who cope maladaptively or have a history of NSSI. The current study's results also suggest a need to develop NHS self‐help resources for ER that can be made widely available (e.g., through their website) as a potential step before primary care involvement (see National Institute for Health and Care Excellence (2011) for their stepped‐care model). Most NHS resources for ER skills focus on children and young people and their families with little targeting towards adults. Considering the need for psychological help among these groups studied, it could help ease caseloads for services and reduce its associated economic impact by helping people earlier.

## 5 Conclusion

NSSI and other maladaptive coping strategies have appeared to be a common phenomenon among a variety of groups and most often with significant impairments. Notwithstanding some limitations, the current study presents a novel attempt to understand the role of ER between historical NSSI behaviours and current coping strategies. It provides meaningful implications for future research and intervention development. This study aimed to focus on adult samples from the general population to further our understanding of these topics compared to the commonly researched paediatric samples. The main results showed that having poor ER mediated the relationship between having a history of NSSI and the use of behavioural disengagement, self‐blame and substance use. Future research considers methodological improvements as well as the need to understand these relationships longitudinally. Clinical implications derived from these findings suggest the importance of continuing ER‐based intervention development and providing earlier access to resources for ER skills. It is hoped the results and insights put forward by this present study can contribute successfully to these fields.

## Conflicts of Interest

The authors declare no conflicts of interest.

## Data Availability

The data that support the findings of this study are available from the corresponding author upon reasonable request.
